# Parotidite aigue néonatale suppurative: à propos de trois cas cliniques avec revue de la littérature

**DOI:** 10.11604/pamj.2016.24.286.10124

**Published:** 2016-07-28

**Authors:** Zineb Isfaoun, Mohammed Amine Radouani, Sihame Azzaoui, Houria Knouni, Hassan Aguenaou, Amina Barkat

**Affiliations:** 1 Centre de Référence de Médecine et de Réanimation Néonatale de l’Hôpital d’Enfants de Rabat, Centre Hospitalier Universitaire Ibn Sina, Avenue Ibn Rochd, Agdal, Rabat, Maroc; 2 Unité Mixte de Recherche en Nutrition et Alimentation URAC 39, Université Ibn Tofail-CNESTEN, RDC-Nutrition AFRA/AIEA, Maroc; 3 Equipe de Recherche en Santé et Nutrition du Couple Mère-Enfant, Faculté de Médecine et de Pharmacie de Rabat, Université Mohammed V de Rabat, Maroc

**Keywords:** Parotidite aiguë suppurative, nouveau-né, traitement, Acute suppurative parotiditis, newborn, treatment

## Abstract

Cet article fait mention de trois cas de parotidite bactérienne néonatale observés pendant une période de sept mois. Ce diagnostic est souvent clinique: on retrouve classiquement hyperthermie, tuméfaction, érythème, chaleur ainsi que sensibilité locale et écoulement purulent au niveau du canal de Sténon lors du massage de la parotide. Le diagnostic clinique est confirmé par échographie et culture de la sécrétion parotidienne purulente. Elles sont le plus souvent d’origine nosocomiale, favorisées par la prématurité et la déshydratation. Traitées précocement, leur évolution est favorable. Les risques liés à l’âge doivent faire débuter une antibiothérapie empirique puis fonction de l'examen direct du pus extrait du canal de Sténon. On isole le plus communément le Staphylocoque aureus; le Streptocoque Viridans et les germes anaérobies. Les parotidites aigues sont très rares en période néonatale: moins de 50 cas ont été rapportés dans la littérature. Nous rapportons trois observations assez particulières. Le diagnostic avait été suspecté sur les signes inflammatoires locaux.

## Abstract (en)

The following case study reports three cases of neonatal bacterial parotiditis observed over a period of seven months. Diagnosis is often based on patient’s clinical features: they typically include hyperthermia, swelling, erythema, warmth as well as local tenderness and purulent discharge at Stensen duct during the massage of the parotid. The clinical diagnosis is confirmed by ultrasound and by culture of parotid purulent secretion. They are mainly of nosocomial origin and are generally caused by prematurity and dehydration. Early treatment improves patient outcome. Risk factors such as age should lead clinicians to start empiric antibiotic therapy first and then antibiotic therapy based on direct examination of pus extracted from Stensen duct. Staphylococcus aureus, Streptococcus Viridans and anaerobic germs are most commonly isolated. Acute parotiditis are extremely rare in the neonatal period: less than 50 cases have been reported in the literature. We here report three rather peculiar cases. The diagnosis was suspected on the basis of signs of local inflammation.

## Introduction

La parotidite aigue bactérienne est rare en période néonatale, moins de 50 observations ont été décrites. Les facteurs prédisposant sont la prématurité, la déshydratation et la présence d’une sonde de gavage. Nous rapportons trois cas de parotidite bactérienne dont un cas compliqué d’abcès parotidien chez trois prématuré entre 35 SA et 36 SA observé au service de néonatologie de l’hôpital d’enfants de Rabat. En présentant nos cas cliniques notre objectif est de rappeler que la parotidite néonatale existe malgré sa rareté, son diagnostic est clinique et une antibiothérapie empirique débutée précocement permet d’éviter les complications.

## Patient et observation

### Observation n°1

**Figure 1:**
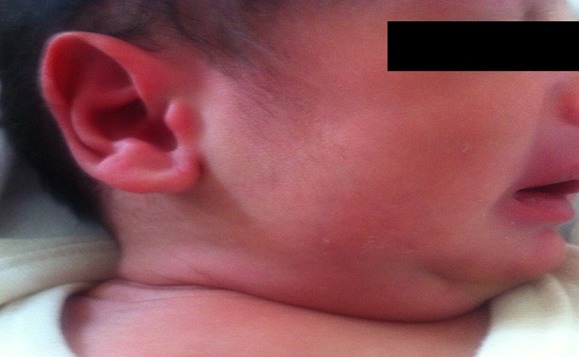
Photo de profil montrant l’augmentation du volume de la loge parotidienne en rapport avec une parotidite

Le premier patient est un nouveau-né à 9 jours de vie qui présente depuis deux jours une fièvre chiffrée à 38,9°C, un refus de tétées avec une augmentation du volume de la loge parotidienne qui est devenu douloureuse à la palpation ce qui a motivé les parents à consulter au pôle des urgences néonatales (Figure 1). C’est un nouveau-né issue d’une grossesse gémellaire suivie au centre de santé, menée à 36 SA, né hypotrophe poids de naissance à 2000 g de sexe masculin. Il n’y avait pas de facteur de risque d’infection materno-fœtale, l’accouchement s’est déroulé par voie basse. La mise au sein juste après l’accouchement avec un allaitement maternel exclusif, la mère a remarqué une chute de poids depuis 4 jours. Il n’a jamais présenté de symptômes auparavant. A l’admission au pôle des urgences néonatales, il était geignard, son état hémodynamique était stable et sa température rectale était à 38,9°C, l’examen clinique trouve une paralysie faciale avec anomalie de fermeture de l’œil droit et une tuméfaction inflammatoire parotidienne droite, douloureuse à la palpation, associée à une perte de poids de 100 g avec une déshydratation légère à 5% et le nouveau-né faisait des pauses respiratoires intermittentes. Il a été hospitalisé immédiatement aux unités de soins intensifs pour la mise en route d’une antibiothérapie par ceftriaxon (100 mg/kg/j) et gentamycine (3mg/kg/j). Cette antibiothérapie avait été poursuivie pendant 48 heures et l’évolution clinique n’était pas satisfaisante d’où le changement de l’antibiothérapie par le ceftazidime et le flucloxacilline avec une bonne évolution. Les examens complémentaires initiaux avaient montré un syndrome inflammatoire modéré avec une leucocytose à 13200 éléments/mm^3^, une protéine C-réactive plasmatique (CRP) à 25 mg/dl initialement puis 51 mg/dl après 48h de traitement passer à 6,7 mg/dl au bout de 48h du changement d’antibiothérapie. L’hémoculture et la culture du liquide céphalo-rachidien et des urines avait été stérile. Le prélèvement bactériologique à l’orifice du canal de Sténon avait révélé la présence d’un Staphylococcus aureus. L’examen clinique de la parotide une semaine après la fin du traitement antibiotique était normal et l’échographie parotidienne ne montrait pas d’anomalie, notamment pas de dilatation canalaire intra-parotidienne. Notant que le deuxième jumeau ne présentait pas de signes cliniques particuliers.

### Observation n°2

**Figure 2:**
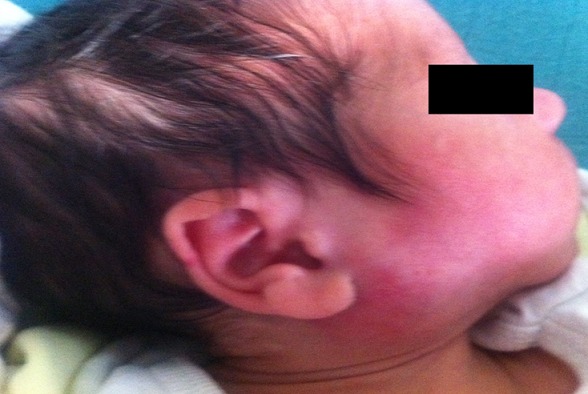
Photo de profil montrant l’aspect clinique d’un abcès parotidien

**Figure 3:**
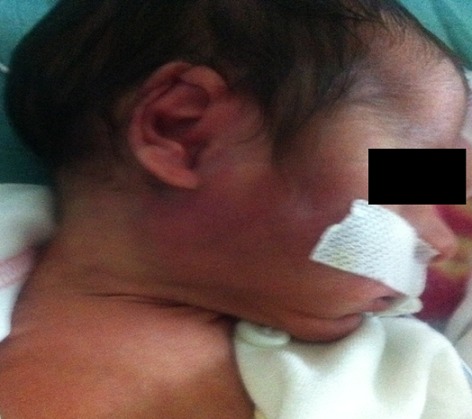
Photo de profil montrant l’évolution de l’abcès parotidien après 48 heures de traitement

Le deuxième cas clinique est un nouveau-né de 23 jours de vie, prématurité de 36 SA, admis dans un tableau de fièvre chiffrée à 40°C avec gonflement de la loge parotide, troubles du comportement et refus de tétées depuis 2 jours. L’examen trouve un nouveau-né geignard avec une masse très inflammatoire au niveau de la loge parotidienne droite avec issu de pus sans signes de compression de la masse. L’échographie a montré la présence d’un abcès parotidien et le bilan inflammatoire était très élevé. La culture du pus trouve un Staphylococcus aureus. L’abcès a été drainé par les chirurgiens et le malade a été mis sous triple antibiothérapie ceftriaxon, flucloxacilline et gentamycine avec une bonne évolution (Figure 2 et Figure 3).

### Observation n°3

Le troisième cas est assez particulier, c’est un nouveau-né à dix jours de vie qui a été admis en néonatologie pour retard de croissance intra-utérin sur prématurité de 35 SA. L’examen à l'admission trouve un nouveau-né avec un poids à 1800 g, rose, tonique, réactif, eupneïque avec une tuméfaction cervicale bilatéral sans fièvre avec une attitude guindée de la nuque. Le malade a été hospitalisé un bilan inflammatoire demandé revenu normal avec une TDM cervicale objectivant une parotidite bilatérale. L’évolution a été marquée par la régression progressive de la tuméfaction sous ceftriaxon et gentamycine. Par ailleurs, le malade présentait aussi des hypoglycémies stabilisés en fin de traitement avec des malformations de l’arbre urinaire: un syndrome de jonction pyélocaliciel et un hypospadias.

## Discussion

Dans nos trois cas clinique, le diagnostic de parotidite aigue suppurative néonatale avait été posé devant la clinique et les caractères inflammatoires de la masse parotidienne. Le diagnostic de cette affection est clinique et pose rarement de problème. Elle est caractérisée par une augmentation du volume avec induration de la glande parotide. La peau en regard est érythémateuse, chaude et douloureuse [1]. La palpation de la glande fait sourdre du pus à l’orifice du canal de Sténon. Le nouveau-né peut être irritable, fébrile. Le diagnostic peut être aidé par l’apport de l’échographie de la glande qui est élargie, hypoéchogène et hyper vascularisée. Les diagnostics différentiels sont la cellulite de la face, l’adénite pré auriculaire ou l’angiome surinfecté. La parotidite aigue bactérienne est une maladie rare en période néonatale. La prévalence estimée par Sabatino et al était de 13,8 cas pour 10000 nouveaux nés hospitalisés en unité de soins intensifs [2]. La parotidite suppurative est majoritairement unilatérale [3], dans notre travail un seul cas parmi les trois patients présentait une parotidite bilatérale. La surinfection de la glande parotide se fait par voie rétrograde par le canal de Sténon. Elle est rarement secondaire d’une anomalie structurelle de la glande parotide ou de son canal. Elle est le plus souvent nosocomiale, principalement décrite dans les unités de soins intensifs néonatals [4]. L’augmentation de la viscosité de la salive est favorisée par la déshydratation responsable d’une obstruction partielle ou par l’alimentation prolongée par sonde gastrique responsable d’une diminution de clairance de la salive [3, 5]. Certaines observations de parotidite compliquée d’un choc infectieux ont fait suggérer que l’infection pouvait se faire également par voie hématogène, les facteurs prédisposant étant la septicémie et l’immunodépression du prématuré [6]. Les examens biologiques sont peu spécifiques, montrant une augmentation des leucocytes à plus de 15000 éléments/mm^3^ prédominant sur les polynucléaires neutrophiles. Comme dans nos observations, le Staphylococcus aureus est le germe le plus souvent responsable de la parotidite aigue suppurée néonatale (65%) [3, 4]. Des souches résistantes «méti-R» ont été isolées dans des observations récentes (2 cas) [7]. D’autres bactéries cocci gram positif ont été rapportées (15%) (Streptocoques Viridans, Streptocoques pyogènes, Staphylocoque à coagulase négatif). Des bacilles gram négatif (15%) (Escherichia coli, Pseudomonas, Klebsielle pneumoniae) ont été identifiés dans des observations de septicémies nosocomiales [3–8]. Les bactéries anaérobies sont plus rares [9]. A l’avenir, l’émergence de souches résistantes de Staphylococcus devra faire reconsidérer le choix thérapeutique initial. Dans la littérature, l’antibiothérapie est instituée par voie intraveineuse et associe une pénicilline M (Cloxacilline, Oxacilline) ou une céphalosporine de troisième génération à un aminoside. La durée du traitement n’est pas consensuelle [10, 11]. Classiquement, dans le cas de Staphylococcus aureus, elle est maintenue par voie intraveineuse entre 7 et 14 jours. En l’absence de syndrome septique, dans certaines observations, un relais oral est pris dès le troisième jour pour une durée de 10 jours [3, 12]. L’antibiothérapie est prolongée chez le prématuré, en cas de défaillance d’autres organes ou lorsqu’un germe anaérobie a été isolé. L’évolution est rapidement favorable avec une diminution du volume de la glande parotide en 24 à 48 heures et une guérison dans 80% des cas sous traitement antibiotique seul [3, 4]. Les complications sont rares. Elles incluent l’abcès intra parotidien, la paralysie faciale, la fistule salivaire et la septicémie. Il n’y a pas eu de complications sévères, ni de décès rapportés depuis 1970. Le drainage chirurgical de la glande reste indiqué dans l’abcès intra parotidien. Il est plus fréquent dans les retards au diagnostic et lorsque le germe est résistant au traitement empirique initial [7, 13].

## Conclusion

La parotidite aigue suppurative néonatale est rare, son diagnostic est clinique. Sous traitement antibiotique précoce, l’amélioration est considérable. Le traitement chirurgical est réservé aux formes compliquées d’abcès. Le pronostic est devenu bon depuis l’avènement des antibiotiques mais la récidive reste possible [14].
